# Human Brain Lipidomics: Pilot Analysis of the Basal Ganglia Sphingolipidome in Parkinson’s Disease and Lewy Body Disease

**DOI:** 10.3390/metabo12020187

**Published:** 2022-02-18

**Authors:** Aaron W. Beger, Beatrix Dudzik, Randall L. Woltjer, Paul L. Wood

**Affiliations:** 1 Anatomy Department, DeBusk College of Osteopathic Medicine, Lincoln Memorial University, Cumberland Gap Pkwy, Harrogate, TN 37752, USA; beatrixjinx@gmail.com; 2 Department of Neurology, Oregon Health & Science University, Portland, OR 97239, USA; woltjerr@ohsu.edu; 3 Metabolomics Unit, College of Veterinary Medicine, Lincoln Memorial University, Cumberland Gap Pkwy, Harrogate, TN 37752, USA; paul.wood@lmunet.edu

**Keywords:** ceramide, dihydroceramide, sphingomyelin, sulfatide, sphingolipid, alpha synuclein, striatum, globus pallidus

## Abstract

Sphingolipids constitute a complex class of bioactive lipids with diverse structural and functional roles in neural tissue. Lipidomic techniques continue to provide evidence for their association in neurological diseases, including Parkinson’s disease (PD) and Lewy body disease (LBD). However, prior studies have primarily focused on biological tissues outside of the basal ganglia, despite the known relevancy of this brain region in motor and cognitive dysfunction associated with PD and LBD. Therefore electrospray ionization high resolution mass spectrometry was used to analyze levels of sphingolipid species, including ceramides (Cer), dihydroceramides (DHC), hydoxyceramides (OH-Cer), phytoceramides (Phyto-Cer), phosphoethanolamine ceramides (PE-Cer), sphingomyelins (SM), and sulfatides (Sulf) in the caudate, putamen and globus pallidus of PD (*n* = 7) and LBD (*n* = 14) human subjects and were compared to healthy controls (*n* = 9). The most dramatic alterations were seen in the putamen, with depletion of Cer and elevation of Sulf observed in both groups, with additional depletion of OH-Cer and elevation of DHC identified in LBD subjects. Diverging levels of DHC in the caudate suggest differing roles of this lipid in PD and LBD pathogenesis. These sphingolipid alterations in PD and LBD provide evidence for biochemical involvement of the neuronal cell death that characterize these conditions.

## 1. Introduction

Parkinson’s disease (PD) is the second most common neurodegenerative disorder, behind only Alzheimer’s disease, with prevalence ranging between 100–200 per 100,000 people [1,2]. PD begins primarily as a hypokinetic movement disorder marked by extrapyramidal motor symptoms, including shuffling gait, bradykinesia, postural rigidity, and facial masking, which progresses to include cognitive dysfunction in 80% of patients within 20 years of diagnosis (i.e., Parkinson’s disease dementia) [3,4]. Meanwhile, Lewy body disease (LBD) is the second most common neurodegenerative dementia following Alzheimer’s disease [5,6]. Onset of LBD is marked by cognitive deficits in the form of dementia, visuospatial difficulties, and fluctuations in executive functioning that can either precede or coincide with extrapyramidal motor symptoms [7,8]. This overlapping spectrum of motor and cognitive involvement between PD and LBD reflects their shared underlying etiology as proteinopathies, exhibiting abnormal deposition of misfolded a-synuclein protein aggregates known as Lewy bodies [7,8].

Lipidomic studies have worked to elucidate the biochemical foundations of these neurodegenerative diseases [9,10]. Of increasing relevance are sphingolipids, a diverse class of structural lipids enriched in neuronal membranes that function to modulate a variety of neurological cell signaling pathways, including neuroinflammation, apoptosis and synaptic transmission [11,12,13]. Altered levels of ceramide, a sphingolipid moderator of apoptosis, have been demonstrated in the plasma and cerebral spinal fluid in PD [10,14,15] and LBD subjects [10,16,17], while sphingolipid enriched domains known as lipid rafts are associated with the localization of a-synuclein along synaptic terminals [18]. Further, glucocerebrosidase, an enzyme involved in sphingolipid metabolism, is altered in the substantia nigra of PD subjects [19] and frontal cortex of LBD subjects [20], promoting cellular susceptibility to a-synuclein aggregation [21]. Significant alteration of sphingolipid levels have also been described in the frontal cortex, visual cortex, anterior cingulate gyrus and substantia nigra of PD and LBD subjects [22,23,24]. 

The basal ganglia constitute a collection of subcortical nuclei that influence motor and cognitive processes, with degradation of this region being well-established in PD and LBD [25,26,27]. Loss of ~80% of the dopaminergic neurons in the nigrostriatal pathway (projections from the substantia nigra pars compacta to the striatum [i.e., putamen + caudate nucleus]) precipitates extrapyramidal motor symptoms [28]. Further, this depletion of dopamine leads to decreased and increased activity of striatopallidal fibers on the internal and external segments of the globus pallidus, respectively [29,30]. Meanwhile, disruption of the frontostriatal dopamine network (circuitous projections between frontal lobe and striatum) yield LBD and PD-related cognitive deficits [31].

Despite involvement in motor and cognitive extrapyramidal circuitry, lipidomic investigations of the basal ganglia outside of the substantia nigra remain underreported. Therefore, we employed electrospray ionization high resolution mass spectrometry to monitor sphingolipid levels in the caudate, putamen and globus pallidus regions of the basal ganglia. Results from healthy controls were compared to PD and LBD subjects. The observed fluctuations in sphingolipids provide insight in the biochemical dysregulation underpinning these conditions. Lipidomics provides data regarding possible alterations in sphingolipids with structural and/or cell signaling roles [9,10,11] and those that might be biomarkers of neuroinflammation [12,13]. These are valuable data for increasing our understanding of the neurodegenerative processes in PD and LBD and currently are not available for the regions included in this study.

## 2. Results

### 2.1. Ceramides

**Figure 1:**
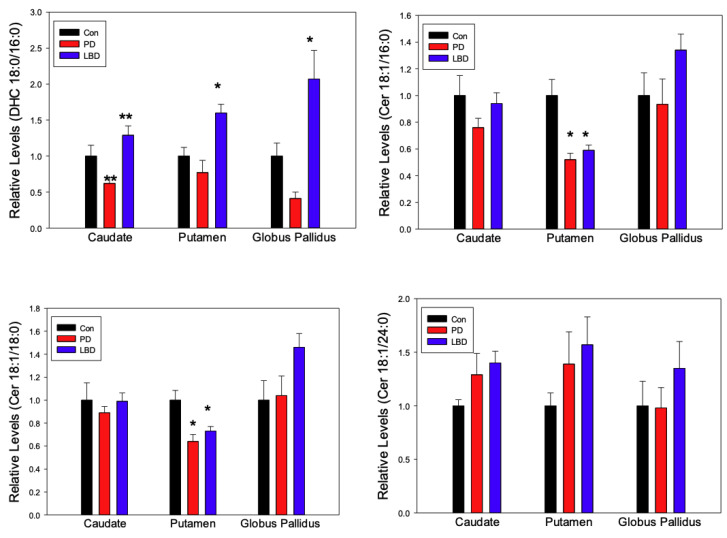
Regional alterations in the relative levels of dihydroceramides (DHC) and ceramides (Cer) in Parkinson’s disease (PD, *n* = 7) and Lewy Body Disease (LBD, *n* = 14). Control (Con, *n* = 9) levels were normalized to 1 ± SEM to simplify the graphical presentation with the PD and LBD levels presented as a fraction or multiple of this value. *, *p* < 0.01 vs. Controls; **, *p* < 0.05 vs. Controls.

**Figure 2:**
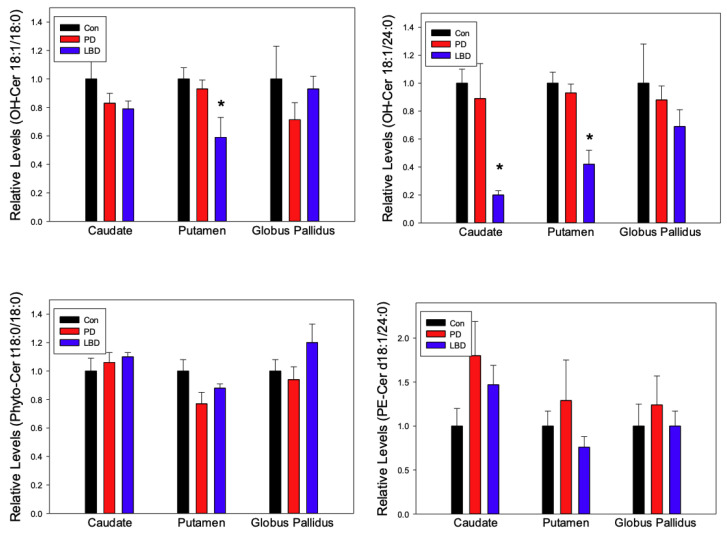
Regional alterations in the relative levels of hydroxyceramides (OH-Cer), phytoceramides (Phyto-Cer), and phosphoethanolamine ceramides (PE-Cer) in Parkinson’s disease (PD, *n* = 7) and Lewy Body Disease (LBD, *n* = 14). Control (Con, *n* = 9) levels were normalized to 1 ± SEM to simplify the graphical presentation with the PD and LBD levels presented as a fraction or multiple of this value. *, *p* < 0.01 vs. Controls.

Chloride adducts [M + Cl]^−^ were monitored as these have been shown to provide the greatest sensitivity for ceramide families [32]. Ceramides (Cer), dihydroceramide (DHC), hydroxyceramides (OH-Cer), phytoceramide (Phyto-Cer) and phosphoethanolamine ceramides (PE-Cer) were monitored in the caudate, putamen, and globus pallidus of control, Lewy body dementia (LBD), and Parkinson’s disease (PD) subjects (Figure 1 and Figure 2). 

Compared to healthy controls, levels of DHC 16:0 were significantly elevated in caudate, putamen and globus pallidus of LBD subjects (Figure 1). Conversely, this lipid was decreased in PD subjects, though the only significant alteration was in the caudate. 

Levels of Cer 16:0 and 18:0 were decreased in the putamen of both LBD and PD subjects (Figure 1), consistent with prior reports of decreased ceramide levels in the anterior cingulate cortex in PD [22]. In contrast, increased ceramide levels have been reported for the visual cortex in PD [33], while no alterations in ceramide levels were observed in the substantia nigra [23].

Hydroxy ceramide (OH-Cer) 18:0 and OH-Cer 24:0 were generally decreased across all three regions in both LBD and PD groups. However, the only significant differences were in the putamen (OH-Cer 18:0 and 24:0) and caudate (OH-Cer 24:0) of the LBD group (Figure 2).

Phyto-Cer is a product of DHC metabolism that has been shown to have a neuroprotective influence in rodents [34,35]. PE-Cer, meanwhile, is an under studied structural analog of sphingomyelin produced in the mammalian plasma membrane [36,37]. Both Phyto-Cer 18:0 and PE-Cer 24:0 did not have significant alterations in this study, however a general increase of PE-Cer 24:0 was noted in the PD group across all three regions. (Figure 2). 

### 2.2. Sphingomyelins (SM)

**Figure 3:**
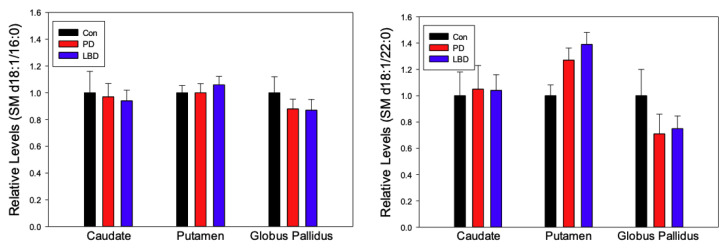
Regional relative levels of sphingomyelins (SM) in Parkinson’s disease (PD, *n* = 7) and Lewy Body Disease (LBD, *n* = 14). Control (Con, *n* = 9) levels were normalized to 1 ± SEM to simplify the graphical presentation with the PD and LBD levels presented as a fraction or multiple of this value.

Levels of SM 16:0 were largely unchanged across the basal ganglia of PD and LBD when compared to healthy controls (Figure 3). The putamen and globus pallidus demonstrated a slight increase and decrease of SM 22:0, respectively, in both LDP and PD, but these alterations were found not found to be significant. (Figure 3). While prior reports have reported decreased levels of SM in the anterior cingulate cortex [22], and increased levels in the substantia nigra [23] and primary visual cortex [33] of PD subjects, our results support those of Gegg et al., who found no SM alterations in the putamen and cerebellum [38]. 

### 2.3. Sulfatide (Sulf)

**Figure 4:**
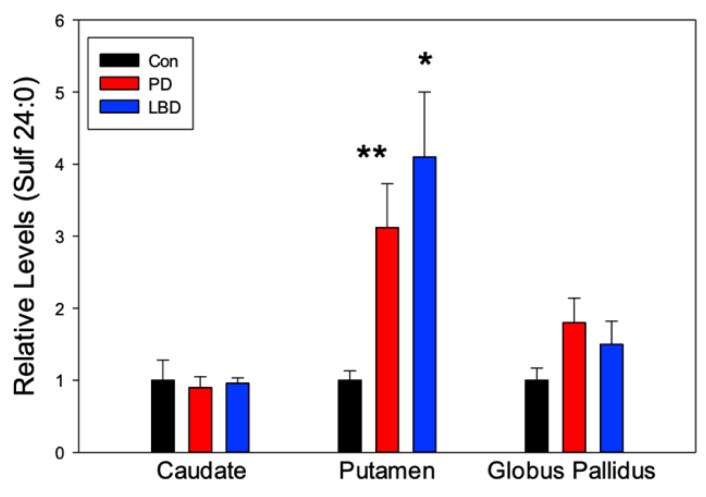
Regional alterations in the relative levels of sulfatide 24:0 (Sulf) in Parkinson’s disease (PD, *n* = 7) and Lewy Body Disease (LBD, *n* = 14). Control (Con, *n* = 9) levels were normalized to 1 ± SEM to simplify the graphical presentation with the PD and LBD levels presented as a fraction or multiple of this value. *, *p* < 0.01 vs. Controls; **, *p* < 0.05 vs. Controls.

Significant elevations in the levels of Sulf 24:0 were observed in the putamen of both PD and LBD subjects (Figure 4), consistent with previous PD findings in the substantia nigra [23] and visual cortex [33].

### 2.4. Gender, Age and PMI

No differences in relative lipid levels were monitored based on gender, age, or PMI. However, this is not unexpected based upon the small sample size and requires validation in a larger sample set.

## 3. Discussion

This is the first detailed analysis of the sphingolipidome in the extrapyramidal system (caudate, putamen, and globus pallidus) in PD and LBD. Lipidomics provides data regarding possible alterations in sphingolipids with structural and/or cell signaling roles [9,10,11] and those that might be biomarkers of neuroinflammation [12,13]. These are valuable data for increasing our understanding of the neurodegenerative processes in PD and LBD and previously were not available for the regions included in this study.

**Figure 5:**
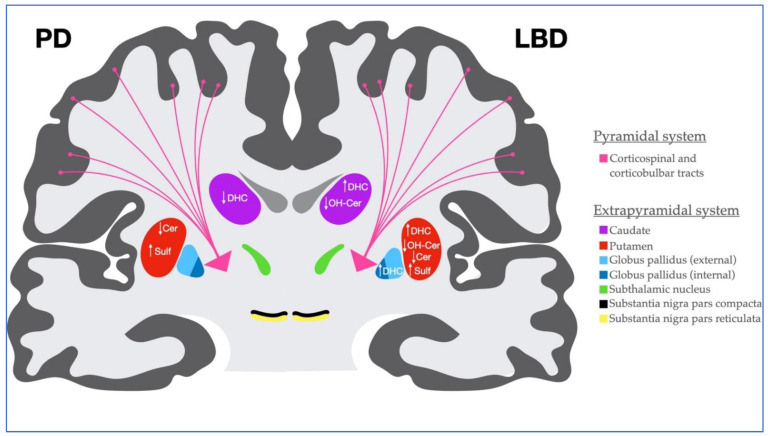
Illustration of coronal section through the brain and midbrain. Topography of the pyramidal and extrapyramidal systems is depicted. Significant lipid alterations monitored in this study for Parkinson’s disease (PD) are located on the left side of the image, and Lewy body disease (LBD) on the right. *Cer, ceramide; DHC, dihydroceramide; OH-Cer, hydroxyceramide, Sulf, sulfatide.*

The observed sphingolipid alterations in the basal ganglia of Parkinson’s disease (PD) and Lewy body disease (LBD) subjects are regionally depicted and summarized in Figure 5. These findings collectively support the growing evidence for the biochemical role of disrupted lipid homeostasis in these synucleinopathies [9,10]. Given the structural and functional roles of these biomolecules in the central nervous system, fluctuations in their levels identified herein is likely a reflection of cell death and synaptic dysfunction in the basal ganglia circuity that is characteristic of PD and LBD. 

Dihydroceramide (DHC), a bioactive precursor to ceramide once believed to be inert, has recently shown relevance in cellular autophagy and oxidate stress signaling pathways [39]. DHC is converted into ceramide via dihydroceramide desaturase (DES1). Inhibition of this enzyme has been shown to elevate DHC levels, promoting autophagic clearing of amyloid-β in neurons, the primary component of amyloid plaques found in the brains of Alzheimer’s patients [39,40]. The observed overall degradation of DHC in PD subjects may then be evidence of depressed autophagy within neurons and microglia, potentially promoting the accumulation of cellular debris, including a-synuclein [41]. Aggregation of a-synuclein secondary to malfunctioning autophagy has been demonstrated in models of PD, further supporting this notion [42]. 

Conversely, LBD subjects exhibited elevated levels of DHC (Figure 1 and Figure 5). This may be a reflection of DHC’s role in the cellular response to oxidative stress in neurodegeneration [39,43]. Depletion of DES1, and subsequent elevation of DHC 16:0 in response to oxidative stress has been demonstrated in mouse hearts and human cell lines [44,45,46]. However, reduced levels of 8-oxo-7,8-dihydro-2’-deoxyguanosine, a marker for DNA-related oxidative damage, have been demonstrated in the caudate and putamen of LBD subjects, complicating this picture and highlighting the need for further study into DHC’s role in LBD [47].

While DHCs were altered in both PD and LBD, no changes in phytoceramides were detected. These data suggest that phytosphingosine may be more important than DHCs for the synthesis of these lipids.

**Figure 6:**
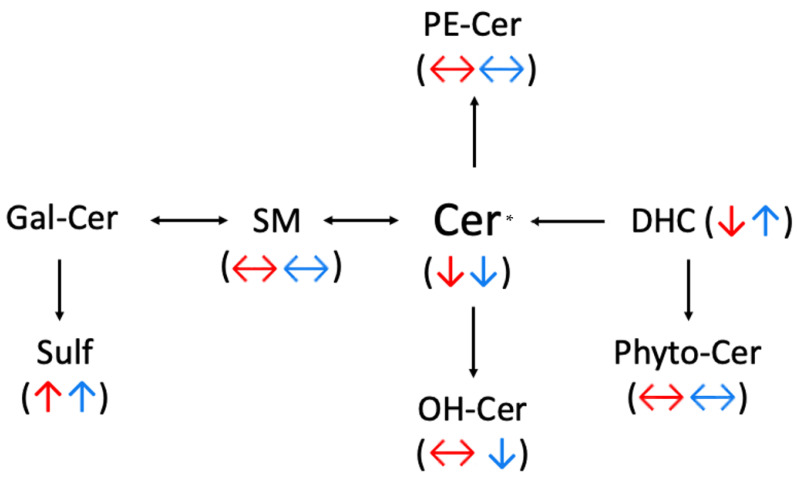
Abbreviated schematic depicting sphingolipid metabolic pathways. Observed significant alterations in relative lipid levels in the putamen are represented by red (Parkinson’s disease) and blue (Lewy body disease) arrows. Colored side arrows indicate no significant alterations were observed in the corresponding condition. *, the decrements in ceramides were restricted to ceramides with short-chain fatty acids. Cer, ceramide; DHC, dihydroceramide; Gal-Cer, galactosyl ceramide; OH-Cer, hydoxyceramide; PE-Cer, phosphoethanolamine ceramide; Phyto-Cer, phytoceramide; SM, sphingomyelin; Sulf, sulfatide.

As the metabolic hub of sphingolipid synthesis (Figure 6), ceramides (Cer) have generated great interest because of their role in neurodegeneration [48,49,50]. Our results indicated a decrease in the levels of ceramides with shorter acyl chains (Cer16:0 and Cer18:0), particularly in the putamen (Figure 1). This is likely reflective of shorter chain ceramides favoring proapoptotic pathways [51]. As PD symptoms first appear with ~80% loss of dopaminergic neurons, the observed depletion of Cer16:0 and Cer18:0 in the striatum of PD subjects may be evidence of the dopaminergic neuronal cell death that has already occurred [52]. Higher levels of these lipids in the striatum may be predicted in prodromal stages of the disease, precipitating dopamine loss and symptom onset. Nonsignificant elevations of the neuroprotective Cer24:0 were also observed, possibly a sign of the surviving neurons abating apoptosis [51]. Although ceramides in the anterior cingulate cortex of PD subjects have been described as being decreased overall [22], inspection of these results reveals increased levels of Cer16:0 and 18:0, and a decrease of Cer24:0 [22], contradicting our results. This suggests the disruption of ceramide homeostasis in PD may be region-dependent and possibly influenced by the length of the acyl chain.

Genetic mutations linked to PD may further explain our observed alterations of ceramide levels. Acid b-glucosidase (GCase), an enzyme encoded by *GBA1*, is responsible for converting glucosylceramide into ceramide and glucose as part of the salvage pathway of ceramide production [53]. *GBA1* mutations, the most common genetic risk factor for PD [54], thus lead to an accumulation of glucosylceramide and reduction in ceramide. This disrupted homeostasis has been linked to a-synuclein aggregation [55,56]. One possible mechanism behind this is that the decreased ceramide levels lead to downregulation of protein phosphatase 2A [57], a key modulator of autophagic pathways, leading to accumulation of a-synuclein [58]. Also implicated with PD is *LRRK2*, with mutations of this gene representing an estimated ~7% of familial cases of PD [59], and 1–2% of sporadic cases [53,60]. Increased activity of GCase has been demonstrated in a *LRRK2^−/−^* transgenic mouse model, leading to increased levels of Cer18:0 in the brain [61], conflicting with the results described here. In addition to the possibility of our subjects having no such mutation of *LRRK2*, this discrepancy may also be linked to the studied brain region. While these authors did not state which brain region they investigated [61], other research has shown the dopaminergic nigrostriatal pathway to be spared in *LRRK2* knockout mice [62]. This suggests that even if our subjects had a *LRRK2* mutation, its influence may have been overshadowed by the augmented lipid levels related to the death of dopaminergic neurons as described above.

Hydroxylation of ceramide is catalyzed by fatty acid 2-hydroxylase to produce OH-Cer. There is scarce data on the involvement of hydroxyceramide (OH-Cer) in LBD and PD. It is possible that the correlation of OH-Cer depletion with Lewy body deposition may be further evidence of the cell death that has occurred, as OH-Cer species have been shown to have a greater apoptotic influence on certain cell types compared to their non-hydroxylated counterparts [63,64]. The monitoring of elevated OH-Cer levels in the prodromal stages of neurological disease may support this observation.

Sphingomyelin (SM) is the most abundant sphingolipid in eukaryotic cells and a major constituent of the myelin sheath [9]. Lewy bodies have been shown to include SM, and SM accumulation due to dysregulation of sphingomyelinase-1 has been associated with PD pathogenesis [65,66]. The frontal cortex of PD subjects has also exhibited altered levels of diacylglycerols, a product of SM metabolism, further implicating SM in PD [10]. Given this, this report’s observations of nonsignificant alterations of SM in the basal ganglia of LBD and PD subjects were unexpected, yet consistent with other lipidomic studies of the putamen [38]. Altered SM levels in the anterior cingulate cortex [33], primary visual cortex [22], and substantia nigra [23] of PD subjects suggest that SM metabolism may be uniquely preserved or imperceptibly augmented in the striatum and globus pallidus.

Sulfatides are enriched in the myelin sheath and function as key mediators of neuroinflammation through glial activation [67]. Depleted activity of arylsulphatase A (ASA), the enzyme responsible for hydrolyzing sulfatide, has been associated with essential tremor in PD patients, an extrapyramidal symptom [68]. ASA malfunction could consequently cause the elevated sulfatide levels observed in the substantia nigra [23] and the striatum and globus pallidus reported herein, leading to the microglial-mediated neuroinflammation observed across the basal ganglia [69].

While this study provided novel insight into the association of PD and LBD and the basal ganglia sphingolipidome, extrapolation of the results should be tempered due to our limited sample size. Employing larger sample sizes in future studies could not only strengthen results, but also control for nuisance variables such as age, sex, therapeutic interventions, and medical history. Further, we did not analyze the internal and external segments of the globus pallidus separately. Given their differing targets and functions within the basal ganglia circuitry, each having a unique lipid profile is plausible.

## 4. Materials and Methods

### 4.1. Ethical Approval

This study was conducted in accordance with the Declaration of Helsinki, and the protocol was approved by the Lincoln Memorial University Institutional Review Board (Ref.# 998V.0).

### 4.2. Human Brain Samples

Brains samples were sourced from the Oregon Brain Bank. Subjects were evaluated and diagnosed by neuropathologists at Oregon Health Sciences University. Brain tissue was obtained via autopsy of volunteer subjects who willfully and ethically donated their tissue, with informed consent provided premortem by the individual, or postmortem by the next-of-kin. Dissections of basal ganglia samples were performed by the neuropathologist (RJW). Following dissection, samples were flash frozen at −80 °C for the biochemical study described here. Demographic information for all individuals used in this study can be found in Table 1.

### 4.3. Lipid Extraction and Analysis

1 mL of methanol, 1 mL of distilled H_2_O and 100µL of stable isotope internal standards were introduced to each sample prior to dismemberment via sonication. 2 mL of methyl-tert-butyl ether was then added, followed by shaking and centrifugation (4000× *g*), each for 30 min at room temperature. 1 mL of organic extract was added to 96-well plates and dried overnight via centrifugal vacuum evaporation. Samples were redissolved in 200µL of infusion solvent (160 mL 2 propanol, 80 mL methanol, 40 mL chloroform, 1 mL H_2_0, 75 mg NH_4_Cl) and subsequently centrifuged at 4000× *g* for 15 min to precipitate any residual particulates [70]. High-resolution (<3 ppm mass error) constant infusion electrospray ionization (ESI) (10µL/min) was performed using an orbitrap mass spectrometer (Thermo Q Exactive). Between each injection, syringe and solvent line were washed with 1 mL of methanol and 1 mL of [hexane: ethyl acetate: chloroform] (3:2:2), respectively, to limit memory effects, as validated by blank injections [32,70].

Samples were scanned first in positive-ESI (280–1800 amu) then negative-ESI (280–1800 amu), each for 0.5 min and at a resolution of 140,000. In negative-ESI, the anions of sphingomyelins were monitored using 5 nmol PC (34:1) [D31] ([M + Cl]^−^ = 824.7359) as a stable isotope internal standard; ceramides were monitored using 1 nmol Cer 16:0 [C16] ([M + Cl]^−^ = 588.5356) as a stable isotope internal standard; dihydroceramides, phytoceramides, phosphoethanolamine ceramides, and hydroxyceramides were monitored using 1 nmol Cer 16:0 [C16] ([M + Cl]^−^ = 588.5356); and sulfatides were monitored using 2 nmol MAG 18:1 [D5] ([M + Cl]^−^ =396.2939). The selection of internal standards was based on detection with <2 ppm mass error and the peak intensities being in the range of the peak intensities of the endogenous lipids. Internal standards with peak intensities that are small fractions or large multiples of the endogenous lipids generate large errors.

The dominant sphingolipids with an RSD of less than 50% were selected for presentation in the graphs. The exact masses for the endogenous lipids are presented in Table 2, based on Lipid Maps (lipidmaps.org) along with the masses for the chloride adducts [32]. All reported lipids were monitored with less than 2 ppm mass error (most were less than 1 ppm) and a peak intensity of at least 200,000 counts. In addition, all lipids were validated by MS/MS [32] and the chloride adducts increase the specificity for the detection of this class of lipids [32].

The raw data is presented in Supplementary Table S1.

### 4.4. Statistical Analysis

For the monitored lipids with <3 ppm mass error, R values were calculated as a ratio of the ion intensity of the endogenous lipid to that of the assigned internal standard. R values were used to calculate the relative standard deviation (RSD = (SD/mean) × 100) for each lipid species, in each brain region, and for each condition (control, PD, and LBD). Lipids with an RSD <~50 were selected for further analysis. Control means were normalized to 1 ± SEM, with PD and LBD levels calculated as a fraction or multiple of this value. One-way analyses of variance (ANOVA) with posthoc Tukey tests were performed to determine the identify significant differences in relative lipid quantities between control samples and PD and LBD samples.

ANOVAs were performed in SPSS (Version 26.0). All other calculations were performed in Microsoft Excel (Version 16.50).

## 5. Conclusions

The sphingolipid profiles of the caudate, putamen and globus pallidus were found to be largely augmented in Parkinson’s disease (PD) and Lewy body disease (LBD) subjects. Alterations of dihydroceramides could be indicative of this lipid’s role in signal transduction pathways related to cellular autophagy and oxidative stress. Levels of ceramides and hydroxyceramides, bioactive sphingolipids with known relevancy in apoptotic pathways, were shown to be modified in both PD and LBD. This is likely a reflection of the loss of dopaminergic neurons in this region. While sphingomyelin species were largely constant across the basal ganglia in both proteinopathies, this lipid has been associated with a-synuclein aggregates, with altered levels having been demonstrated in other brain regions. Stability of sphingomyelin metabolism in PD and LBD may therefore be unique to the basal ganglia. Sulfatides were significantly increased in PD and LBD, a sign of the neuroinflammation associated with these conditions.

In summary we have utilized high-resolution mass spectrometry to characterize the sphingolipidome of the extrapyramidal system. Our study has added to our database of complex lipid changes that may be involved in the neurodegenerative processes that underlie PD and LBD. The limitation of our study is that it is a probe study of a small sample set and requires validation in a much larger study which should include longitudinal sampling.

## Figures and Tables

**Table 1:** Demographic information for formalin fixed, control, Parkinson’s disease (PD) and Lewy body dementia (LBD) subjects.

Parameter	Controls	PD	LBD
*n*	9	7	14
Age (Yrs ^†^ ± SD) [Range]	68.25 ± 11.06 [52–81]	88.83 ± 7.11 [78–100]	81.42 ± 9.47 [64–93]
PMI (Hrs */Yrs ^†^ ± SD) [Range]	17.75 ± 4.46 [11–23]	18.88 ± 19.1 [2–52]	10.13 ± 8.58 [3–32]
Sex (M:F)	5:3	4:3	9:5
Neuritic plaques (sparse) *n*	2	3	11
Neuritic plaques (moderate) *n*	0	1	3
Tangles Braak 1 *n*	5	3	2
Tangles Braak 2 *n*	0	1	5
Tangles Braak 3 *n*	0	0	1
Tangles Braak 4 *n*	0	3	6
Cortical Lewy Bodies *n*	0	7	14

* Hrs, hours; LBD, Lewy body dementia; PD, Parkinson’s disease; PMI, post-mortem interval; SD, standard deviation; ^†^ Yrs, years.

**Table 2:** Masses and ions selected for monitoring the reported sphingolipids.

Lipid	Exact Mass	[M + Cl]^−^
DHC d18:0/16:0	539.5272	574.4975
Cer d18:1/16:0	537.5121	572.4819
Cer d18:1/18:0	656.5434	600.5132
Cer d18:1/24:0	649.6373	684.6071
OH-Cer d18:1/18:0	581.5382	616.5082
OH-Cer d18:1/24:0	665.6322	700.6021
PhytoCer t18:0/18:0	583.5540	618.5238
PE-Cer d18:1/18:0	772.6458	807.6156
SM d18:1/16:0	704.5832	739.5531
SM d18:1/22:0	788.6771	823.6470
Lipid	Exact Mass	[M−H]^−^
Sulf d18:1/24:0	863.6156	862.6082

## Data Availability

Raw data is available as a Supplementary Table S1.

## Supplementary Materials

The following are available online at https://www.mdpi.com/article/10.3390/metabo12020187/s1, Table S1: Raw data.
